# Accounting for treatment use when validating a prognostic model: a simulation study

**DOI:** 10.1186/s12874-017-0375-8

**Published:** 2017-07-14

**Authors:** Romin Pajouheshnia, Linda M. Peelen, Karel G. M. Moons, Johannes B. Reitsma, Rolf H. H. Groenwold

**Affiliations:** 1 Julius Center for Health Sciences and Primary Care, University Medical Center Utrecht, PO Box 85500, 3508 GA Utrecht, the Netherlands; 2 Cochrane Netherlands, University Medical Center Utrecht, Utrecht, The Netherlands

## Abstract

**Background:**

Prognostic models often show poor performance when applied to independent validation data sets. We illustrate how treatment use in a validation set can affect measures of model performance and present the uses and limitations of available analytical methods to account for this using simulated data.

**Methods:**

We outline how the use of risk-lowering treatments in a validation set can lead to an apparent overestimation of risk by a prognostic model that was developed in a treatment-naïve cohort to make predictions of risk without treatment. Potential methods to correct for the effects of treatment use when testing or validating a prognostic model are discussed from a theoretical perspective.. Subsequently, we assess, in simulated data sets, the impact of excluding treated individuals and the use of inverse probability weighting (IPW) on the estimated model discrimination (c-index) and calibration (observed:expected ratio and calibration plots) in scenarios with different patterns and effects of treatment use.

**Results:**

Ignoring the use of effective treatments in a validation data set leads to poorer model discrimination and calibration than would be observed in the untreated target population for the model. Excluding treated individuals provided correct estimates of model performance only when treatment was randomly allocated, although this reduced the precision of the estimates. IPW followed by exclusion of the treated individuals provided correct estimates of model performance in data sets where treatment use was either random or moderately associated with an individual's risk when the assumptions of IPW were met, but yielded incorrect estimates in the presence of non-positivity or an unobserved confounder.

**Conclusions:**

When validating a prognostic model developed to make predictions of risk without treatment, treatment use in the validation set can bias estimates of the performance of the model in future targeted individuals, and should not be ignored. When treatment use is random, treated individuals can be excluded from the analysis. When treatment use is non-random, IPW followed by the exclusion of treated individuals is recommended, however, this method is sensitive to violations of its assumptions.

**Electronic supplementary material:**

The online version of this article (doi:10.1186/s12874-017-0375-8) contains supplementary material, which is available to authorized users.

## Background

Prognostic models have a range of applications, from risk stratification, to use in making individualized predictions to help counsel patients or guide healthcare providers when deciding whether or not to recommend a certain treatment or intervention [1–3]. Before prognostic models can be used in practice, their predictive performance (e.g. discrimination and calibration)- in short, performance- should be evaluated in a set of individuals who are representative of future targeted individuals. In studies that use independent data to validate a previously developed prognostic model, performance is often considerably worse than in the development set [4]. This may be due to, for example, overfitting of the model in the development data set [5, 6] or differences in case-mix (between the development set and validation sets [7–10].

One aspect that can vary considerably between data sets used for model development and validation is the use of treatments or preventative interventions that affect (reduce) the occurrence of the outcomes under prediction. Although a difference in the use of treatments between a development and validation set is generally viewed as a difference in case-mix characteristics, treatment use in a validation set can actually lead to further problems. When additional treatment use in a validation set (compared to the development set) results in a markedly lower incidence of the outcome under prediction, the predictive performance of the model will likely be affected. A challenge arises when a prognostic model has originally been developed in order to make predictions of “untreated risks”, i.e. predictions of an individual’s prognosis without certain treatments, to guide the decision to initiate those treatments in future targeted individuals. Ideally these models should be validated in data sets in which individuals remain untreated with those specific treatments throughout follow-up, so-called treatment-naïve populations. However, the use of such treatment-naïve populations is uncommon and poor performance of a prognostic model seen in a validation study could be directly attributed to treatment use in the validation data set [11, 12].

Ignoring the effects of treatment use in the *development* phase of a prognostic model for the prediction of untreated risks has already been shown to lead to a model that underestimates this risk in future targeted individuals [13]. However, it is not clear to what extent treatment use in a validation set might influence the observed performance of a prognostic model that was developed in a treatment-naïve population, or how one can account for additional treatment use in a validation set in order to correctly estimate how a prognostic model would perform in its target (untreated) population using a treated validation set.

In this paper, we provide a detailed explanation of when and how treatment use in a validation set can bias the estimation of the performance of a prognostic model in future targeted (untreated) individuals and compare different analytical approaches to correctly estimate the performance of a model using a partly treated validation data set in a simulation study.

## Methods

### Problems with ignoring treatment use in a validation study

**Fig. 1:**
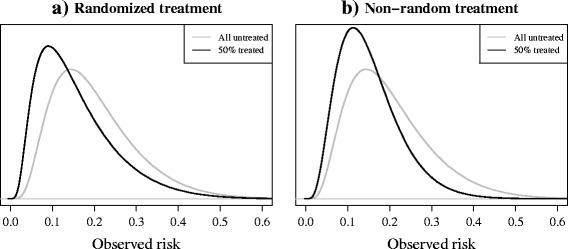
**a-b**: Risk distributions in two simulated validation sets. 50% of individuals received an effective treatment (relative odds reduction on treatment: 0.5), (see Table 2 scenarios 2 and 1, respectively, for details). **a** the model was validated on the combined treatment and control group of a randomised trial. **b** the model was validated using data from a non-randomised setting where the probability of receiving treatment depended on an individual’s (untreated) outcome risk. *Black lines* represent the observed risks in the validation set, after treatment. *Grey lines* represent the risks of the same individuals had they (hypothetically) remained untreated

If individuals in a validation set receive an effective treatment during follow-up, their risk of developing the outcome will decrease. Figs. 1a and b show the effect of treatment use on the distribution of risks in data sets that represent data from a randomized trial (RCT) and a non-randomized study (e.g. routine care data or data from an observational cohort study) in which treatment use was more likely in high-risk individuals. In the event of the use of an effective treatment, fewer individuals will develop the outcome than would have, had they remained untreated, and thus the observed outcome frequencies will be lower than the predicted “untreated” outcome frequencies. As a result, a prognostic model developed for making predictions of risk without that treatment (i.e. models used to guide the initiation of a certain treatment) will erroneously appear to overestimate risk in a partially treated validation set, regardless of how treatments have been allocated. As the aim, in this case, is to estimate the performance of the model when used for future, untreated individuals, measures of model discrimination and calibration will give a biased representation of the performance of the model when used in practice for making untreated outcome predictions, if treatment use in the validation set is ignored.

The effect that treatment use will have on measures of model performance in a validation study will depend on a number of factors, including the strength of the effect of treatment on the outcome risk, the proportion of individuals receiving treatment, and the underlying pattern of treatment use. If a treatment has a weak effect on the outcome risk or only a small proportion of individuals are treated in a validation set, the impact on model discrimination and calibration will be relatively small. Furthermore, the way in which treatments are allocated to individuals, whether treatment is allocated randomly, as in data from an RCT, or non-randomly and treatment use is rather based on an individual’s risk-profile or according to strict treatment guidelines, will influence the impact that treatment use will have in a validation study. If, for example, high-risk individuals are selectively treated, we can anticipate an even greater impact of treatment use on measures of model performance. In this case, the distribution of observed risks will become narrower, due to the risk-lowering effects of treatment in the high-risk individuals (see Fig. 1b), making it more difficult for the model to discriminate between individuals who will or will not develop the outcome, and the calibration in high-risk individuals will be most greatly affected.

### Methods to account for treatment use

In this section we describe possible approaches to account for treatment use in a validation study. For each method, the rationale, expected result of its use, and potential issues are outlined. A summary of the methods, including additional technical details can be found in Table 1.

**Table 1:** Possible methods to account for the effects of treatment in a validation set

Approach	Implementation	Key considerations
*1. Exclude treated individuals*	1. Exclude any individual who received treatment between the point of prediction and the assessment of the outcome from the analysis. 2. Estimate model performance in only the untreated subset.	- Provides correct estimates of performance in the (untreated) target population if treatment use is not associated with other prognostic factors.† - Decreases the effective sample size.
*2. Inverse probability weighting*	1. Fit a propensity score (PS) model for treatment in the validation set using logistic regression: logit(Tr_*i*_) = ${\upalpha}_0+{\sum}_{i=1}^{\mathrm{n}}\left({\upalpha}_i{\mathrm{X}}_i\right)$ 2. Calculate PS for individuals using the estimates from the fitted PS model: PS_*i*_ = ${\sum}_{i=1}^{\mathrm{n}}\left({\widehat{\upalpha}}_i{\mathrm{X}}_i\right)$ 3. Calculate inverse probability weights (w_i_) for each untreated individual based on their individual PS: w_*i*_ = 1 / (1 - PS_*i*_) [17] 4. Exclude treated individuals from the analysis set. 5. *(optional)* Truncate weights [21]. 6. Estimate weighted measures of model performance in only the untreated subset.	- Provides correct estimates of performance in (untreated) target population if treatment use is or is not associated with other prognostic factors, provided key assumptions of IPW are met.† - Does not provide correct estimates in the presence of non-positivity, or when there are unobserved predictors that are strongly associated with both the outcome and use of treatment [15, 18]. - Exclusion of treated individuals decreases the effective sample size. - Extreme weights can further reduce precision and introduce bias.
*3. Recalibration*	1. Calculate the linear predictor of the prognostic model: LP0_*i*_ = ${\sum}_{i=1}^{\mathrm{n}}\left({\widehat{\upbeta}}_i{\mathrm{X}}_i\right)$ 2. Re-estimate the model intercept in the full validation data [23, 22]. logit(Y_*i*_) = *γ*_0_ + *offset*(LP0_*i*_) 3. Calculate the updated linear predictor. LP1_*i*_ *=* ${\widehat{\gamma}}_0$ + LP0_*i*_ 4. Estimate model performance using LP1.	- Does not affect discrimination. - Not sufficient to correct calibration if relative treatment effects are heterogeneous or use is associated with an individual’s risk. - Adjusts for other differences in case-mix leading to misleading estimates of the calibration of the original model.
*4. Model treatment*	1. Refit the original prognostic model using the full validation data, including an indicator term for treatment use and treatment interaction terms. i) with recalibration of the intercept: logit(Y_*i*_) = *γ*_0_ + *offset*(LP0_*i*_) + *γ*_*Tr*_Tr_*i*_ * ii) with a full refit of the original model: logit(Y_*i*_) = *γ*_0_ + ${\sum}_{i=1}^{\mathrm{n}}\left({\upgamma}_i{\mathrm{X}}_i\right)$+ *γ*_*Tr*_Tr_*i*_ * 2. Calculate the updated linear predictor. i) LP2_*i*_ = ${\widehat{\gamma}}_0$ + ${\sum}_{i=1}^{\mathrm{n}}\left({\widehat{\upbeta}}_i{\mathrm{X}}_i\right)$+ ${\widehat{\gamma}}_{Tr}$Tr_*i*_ * ii) LP3_*i*_ = ${\widehat{\gamma}}_0$ + ${\sum}_{i=1}^{\mathrm{n}}\left({\widehat{\upgamma}}_i{\mathrm{X}}_i\right)$+ ${\widehat{\gamma}}_{Tr}$Tr_*i*_ * 3. Estimate model performance using LP2 or LP3.	- Can lead to an over-estimation of model discrimination. - Adjusts for other differences in case-mix leading to misleading estimates of the calibration of the original model.

Abbreviations: *X*_*i*_ design matrix (predictor values) for individual *i;* *Y*_*i*_ outcome for individual *i;* *LP* linear predictor; *PS* propensity score; *Tr* treatment

${\widehat{\upalpha}}_i$ represent coefficients of the treatment propensity model for individual *i*

${\widehat{\upbeta}}_i$ represent coefficients of the original prognostic model for individual *i*

${\widehat{\upgamma}}_i$ represent coefficients of the updated prognostic model for individual *i*

*Interaction terms between treatment use and predictors should be included where necessary

^†^Estimates will be correct providing all other modelling assumptions are met

#### Exclusion of treated individuals from the analysis

A common and straightforward approach to remove the effects of treatment is to exclude from the analysis individuals in the validation data set who received treatment. In doing this, one assumes that the untreated subset will resemble the untreated target population for the model.

**Fig. 2:**
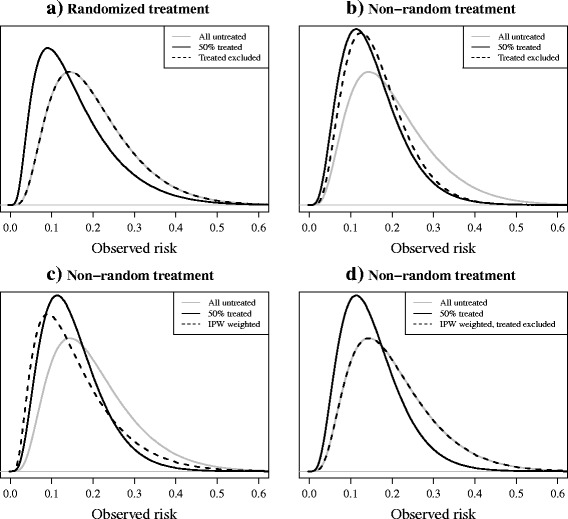
**a-d**: Risk distributions in two simulated validation sets, before and after applying different approaches to correct for treatment use. 50% of individuals received an effective treatment (relative odds reduction on treatment: 0.5) (see Table 2 scenarios 2 and 1, respectively, for details). **a** the model was validated on the combined treatment and control group of a randomised trial. **b**-**d** the model was validated using data from a non-randomised setting where the probability of receiving treatment depended on an individual’s (untreated) outcome risk. *Solid black lines* represent the observed risks in the validation set after treatment. *Dashed black lines* represent the risks observed after applying correction methods to the data: **a**-**b** the exclusion of treated individuals, **c** IPW, **d** IPW followed by the exclusion of treated individuals. *Grey lines* represent the risks of the same individuals had they remained untreated

As Fig. 2a shows, in settings where treatment is randomly allocated (Table 2, scenario 2), the exclusion of treated individuals will result in a validation set that is indeed still representative of the target population. As a result, measures of discrimination and calibration are the same as they would be had all individuals remained untreated, and thus are correct estimates of the performance of the model in its target population.. However, the effective sample size is reduced, (e.g. a 50% reduction in the case of an RCT with 1:1 randomization).

**Table 2:** A summary of fifteen simulated scenarios

Scenario	Specification						
	Data generating models (development and validation sets)†	Sample size of data sets	% outcome (before treatment)	Baseline risk in the absence of treatment (Risk)	Treatment allocation model	% treated in validation set	Treatment effect model
1 (Default)	logit(Y) = −1.50 + 1*X_1_ + 1*X_2_ + 0*U	1000	20	1 / (1 + exp.(1.50–1*X_1_–1*X_2_–0*U))	P(Tr) = 1 / (1 + exp. (1.95–10*Risk))	50	OR_Tr_ = 0.5
2	-	-	-	-	P(Tr) = 0.50	-	-
3	-	-	-	-	-	-	OR_Tr_ = 1 / (1 + exp.(−1 + 5*Risk))
4	-	-	-	-	P(Tr) = 1 / (1 + exp. (18–100*Risk))	-	-
5	-	-	-	-	P(Tr) = 1 / (1 + exp. (3.30–10*Risk))	25	OR_Tr_ = 0.3
6	-	-	-	-	P(Tr) = 1 / (1 + exp. (3.30–10*Risk))	25	-
7	-	-	-	-	P(Tr) = 1 / (1 + exp. (3.30–10*Risk))	25	OR_Tr_ = 0.8
8	-	-	-	-	-	-	OR_Tr_ = 0.3
9	-	-	-	-	-	-	OR_Tr_ = 0.8
10	-	-	-	-	P(Tr) = 1 / (1 + exp. (0.70–10*Risk))	75	OR_Tr_ = 0.3
11	-	-	-	-	P(Tr) = 1 / (1 + exp. (0.70–10*Risk))	75	-
12	-	-	-	-	P(Tr) = 1 / (1 + exp. (0.70–10*Risk))	75	OR_Tr_ = 0.8
13	logit(Y) = −1.55 + 1*X_1_ + 1*X_2_ + 1*U	-	-	1 / (1 + exp.(1.55–1*X_1_–1*X_2_–1*U))	P(Tr) = 1 / (1 + exp. (1.90–10*Risk))	-	-
14	logit(Y) = −1.70 + 1*X_1_ + 1*X_2_ + 2*U	-	-	1 / (1 + exp.(1.70–1*X_1_–1*X_2_–2*U))	P(Tr) = 1 / (1 + exp. (1.80–10*Risk))	-	-
15	logit(Y) = −2.15 + 1*X_1_ + 1*X_2_ + 4*U	-	-	1 / (1 + exp.(2.15–1*X_1_–1*X_2_–4*U))	P(Tr) = 1 / (1 + exp. (1.55–10*Risk))	-	-

Abbreviations: *P(Tr)* probability of treatment; Risk: baseline risk of an individual in the validation set, prior to treatment; *OR*_Tr_ relative effect of treatment on the risk of outcome Y

†Predictors X_1_, X_2_, and U were independent random draws from a normal distribution (mean = 0, variance = 0.2); the binary outcome Y was sampled from a binomial distribution with outcome probability derived from the data generating model

Scenario 1 is the default scenario on which all other scenarios are based. Where cells are empty (“-”), the default parameter value from scenario 1 was used

Figure 2b represents a study where treatment allocation was non-random and high-risk individuals had a higher probability of being treated (Table 2, scenario 1). If treatments were initiated between the moment of making a prediction and the assessment of the outcome, the exclusion of treated individuals results in a subset of individuals with a lower risk on average than in the untreated target population. As a result, the case-mix (in terms of risk profile) in the data set will become more homogenous, and one can expect measures of discrimination to decrease [9, 14], underestimating the true discriminative ability of the model in future targeted individuals. While this approach may appear to provide correct estimates of calibration, the interpretation of these measures is limited due to the inherent selection bias. The non-randomly untreated individuals only represent a portion of the total target population. Hence, estimates of model performance may provide little information about how well calibrated the model is for high-risk individuals, as these have been actively excluded.

#### Inverse probability weighting

An alternative approach for model validation in data sets with non-random treatment use would be to balance the data in such a way that it resembles that of an RCT. Inverse probability weighting (IPW) is a method applied in studies where the aim is to obtain an estimate of the causal association between an exposure and outcome, accounting for the influence of confounding variables on the effect estimate [15]. A “treatment propensity model” is first fitted to the validation data, regressing an indicator (yes/no) of treatment use (dependent variable) on any measured variables that may be predictive of treatment use (independent variables), including the predictors of the prognostic model that is being evaluated [16]. Subsequently this treatment propensity model is then used to estimate for each individual in the validation set the probability of receiving the treatment, based on his/her observed variables (risk profile). Following this, each individual is weighted by the inverse of their own probability of the actual treatment received [17], resulting in a distribution of risks in the validation set that resembles what would have been seen had treatments been randomly allocated, as shown by the similarity of the solid black line in Fig. 2a and the dashed black line in Fig. 2c. By excluding treated individuals after deriving weights, the resulting validation set should resemble the untreated target population, as seen in Fig. 2d. However, this will again result in a smaller effective sample size for the validation.

IPW is subject to a number of theoretical assumptions [15, 18, 19]. One example of a violation of these assumptions is practical non-positivity (i.e. it may be that in some risk strata no subjects received the treatment) [20], which may arise if a subset of individuals has a contraindication for treatment or when guidelines already recommend that individuals above a certain probability threshold should receive treatment. This can lead to individuals receiving extreme weights, resulting in biased and imprecise estimates of model performance [15]. In addition, problems can occur due to incorrect specification of the treatment propensity model, for example due to the presence of unmeasured confounders- predictors associated with both the outcome and the use of treatment in the validation set. Variants of the basic IPW procedure can be applied, such as weight truncation, which may improve the performance of this method in settings where the assumptions are violated [21].

#### Model recalibration

The incidence of the predicted outcome may vary between development and validation data sets. If this is the case, the predictions made by the model will not, on average, match the outcome incidence in the validation data set [22]. As discussed in section 2.1, use of an effective treatment in a validation data set will lead to fewer outcome events and thus a lower incidence than there would have been had the validation set remained untreated. One approach to account for this would be to recalibrate the original model using the partially treated validation data set. In a logistic regression model, a derivative of the incidence of the outcome is captured by the intercept term in the model, and thus a simple solution would seem to be to re-estimate the model intercept using the validation data set [23, 24]. In doing this, the average predicted risk provided by the recalibrated model should then be equal to the (observed) overall outcome frequency in the validation set. Further details of this procedure are given in Table 1. Where treatment has been randomly allocated, intercept recalibration should indeed account for the risk-lowering effects, provided that the magnitude of the treatment effect does not vary depending on an individual’s risk and thus is constant over the entire predicted probability range. In non-randomized settings, where treatment use by definition is associated with participant characteristics, a simple intercept recalibration is unlikely to be sufficient due to interactions between treatment use and patient characteristics that are predictors in the model.

However, although recalibration may seem a suitable solution for modelling the effects of treatment, when applying recalibration, concerns should also be raised over the interpretation of the estimated performance of the model. Differences in outcome incidence between the development data set and validation data set may not be entirely attributable to the effects of treatment use. By recalibrating the model to adjust for differences in treatment use and effects, we simultaneously adjust for differences in case-mix between the development and validation set. As the aim of validation is to evaluate the performance of the original prognostic model, in this case in a treatment-naïve sample, recalibration may actually lead to an optimistic impression of the accuracy of predictions made by the original model in the validation set. For example, if the validation set included individuals with a notably greater prevalence of comorbidities and thus were more likely to develop the outcome, recalibration prior to validation could mask any inadequacies of the model when making predictions in this subset of high-risk individuals. Thus recalibration is not an appropriate solution to the problem.

#### Incorporation of treatment in the model

A more explicit way to deal with treatment use would be to update the prognostic model with treatment use added as a new predictor. If effective, treatment can actually be considered to be a missing predictor in the original developed model. However, unlike other predictors, when validating a model in a non-randomised data set, we cannot know whether a person in practice will indeed receive the treatment at the point of making a prediction. By adding a binary predictor for treatment use to the original prognostic model, one may aim to alleviate the misfit that results from the omission of this predictor, and get closer to the actual performance of the original model in the validation set, had individuals remained untreated.

There are a number of approaches to updating a model with a new predictor [23, 22, 25]. One option would be to incorporate an indicator for treatment on top of the prognostic model, keeping the original model coefficients fixed. However, in doing this we assume that there is no correlation between treatment use and the predictors in the model. Instead the model could be entirely refitted with the addition of an indicator term for treatment using the validation data set (for further details, see Table 1). It may be necessary to include statistical interaction terms in the updated model, where anticipated [26].

A challenge when considering this approach is the correct specification of the updated prediction model. Failure to correctly specify any interactions between treatment and other predictors in the validation set could mean that the effects of treatment are not completely taken into account. Furthermore, the addition of a term for treatment to the model that is to be validated may improve the performance beyond that of the original model due to the inclusion of additional predictive information. Thus, as with recalibration, we do not recommend this approach.

### Outline of a simulation study

We assess the performance of different methods to account for the effects of treatment in fifteen scenarios using simulated data. The effectiveness of two methods described in section 2.2, model recalibration and the incorporation of a term for treatment use in the model, are not present, as their inferiority has already been discussed.

Details of the simulation study are provided in Table 2, which describes 15 scenarios that were studied. For each scenario, a development data set of 1000 individuals of whom all remained untreated throughout the study was simulated. A prognostic model was developed with two predictors using logistic regression analysis, specifying the model so it matched the data generating model. Fifteen validation sets of 1000 individuals were drawn using the same data generating mechanism as their corresponding development data sets, representing an ideal untreated validation set to estimate the model’s ability to predict untreated risks. Subsequently, 50% of the individuals in each validation set were simulated to receive a risk-lowering point-treatment with a constant effect of a reduction in the outcome odds by 50%.

In scenarios 1, 3 and 4, an individual’s probability of receiving treatment was a function of their untreated risk of the outcome, representing observational data. In scenario 2, treatment was randomly allocated to individuals, simulating data from an RCT. In scenarios 1 and 3, there was a moderate positive association between risk and treatment allocation, and thus individuals with a more “risky” profile were more likely to receive treatment. In scenario 4 this association was large: treatment was allocated to most (95%) of the individuals with a predicted risk higher than 18%. In scenario 3, the relative treatment effect was allowed to increase with increasing risk. Using scenario 1 as a starting point, in scenarios 5–12, the effect of treatment on risk varied from strong to weak, and the proportion of individuals treated varied. In scenarios 13–15, an unobserved predictor with varying association (moderate negative, weak positive or strong positive) with the outcome was included in the data generating model.

The performance of the prognostic model was estimated in each of these data sets, first ignoring the effects of treatment, and again either by first excluding treated individuals from the analysis, or by applying IPW methods (as specified in Table 1). We applied standard IPW and IPW with weight truncation (at the 98th percentile). For scenarios 1–12, the treatment propensity model was correctly specified; for scenarios 13–15, the unobserved predictor was (by definition) omitted from the treatment propensity model.

In all simulated validation sets and for all methods being applied, performance was estimated in terms of the c-index (area under the ROC curve) and observed:expected (O:E) ratio. For scenarios 1–4 and 13–15 calibration plots were constructed. For IPW methods, calculated IPW weights were used to estimate weighted statistics (see Additional file 1 for further details). In order to obtain stable estimates of the c-index and O:E ratio, we repeated the process of data generation, model development and validation 10,000 times, calculating the mean and standard deviation (SD) of the distribution of the 10,000 estimates. Calibration plots were based on sets of 1 million individuals (equivalent to combining results from 1000 repeats in data sets with 1000 individuals) for each scenario. R code to reproduce the analyses can be found in Additional file 1.

## Results

**Fig. 3:**
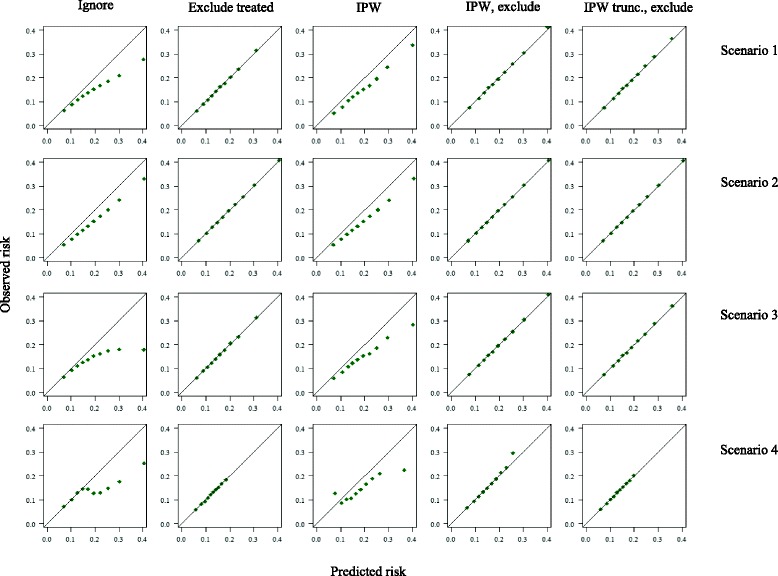
Calibration curves calculated in a treated validation set, following different approaches to account for the effects of treatment. Scenario 1: P(treatment) increases with risk, fixed treatment effect; scenario 2: randomized treatment, fixed treatment effect; scenario 3: P(treatment) increases with risk, treatment effect increases with risk; scenario 4: 18% baseline risk threshold for treatment, fixed treatment effect. Plots were based on sets of 1 million individuals

**Fig. 4:**
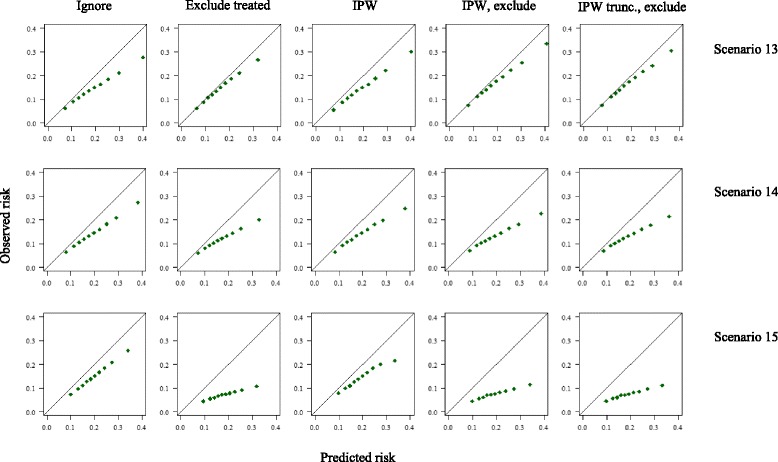
Calibration curves calculated in a treated validation set, following different approaches to account for the effects of treatment, in the presence of an unmeasured predictor (U) associated with both the outcome and the probability of receiving treatment. Scenario 13: U has a weak association with the outcome (log(OR) = 1); scenario 14: U has a moderate association with the outcome (log(OR) = 2); scenario 15: U has a strong association with the outcome (log(OR) = 4). Plots were based on sets of 1 million individuals.

Results of the simulation study are presented below. A summary of the estimated performance measures in each scenario can be found in Tables 3 and 4, and calibration plots for scenarios 1–4 and 13–15 are depicted in Figs. 3 and 4, respectively.

**Table 3:** Estimated calibration in the validation set (observed:expected (O:E) ratio) across fifteen different simulated scenarios

Scenario	Method					
	Reference: untreated	Ignore treatment	Exclude treated	IPW	IPW, exclude	IPW_trunc_ exclude
1	1.00 (0.09)	0.76 (0.07)	1.01 (0.13)	0.79 (0.09)	1.00 (0.13)	1.00 (0.12)
2	1.00 (0.09)	0.79 (0.07)	1.00 (0.11)	0.79 (0.07)	1.00 (0.11)	1.00 (0.11)
3	1.01 (0.09)	0.69 (0.07)	1.00 (0.13)	0.76 (0.09)	1.00 (0.13)	1.00 (0.12)
4	1.00 (0.09)	0.72 (0.07)	1.01 (0.16)	0.74 (0.30)	0.98 (0.44)	1.00 (0.17)
5	1.00 (0.09)	0.80 (0.08)	1.00 (0.13)	0.68 (0.07)	1.00 (0.10)	1.00 (0.10)
6	1.00 (0.09)	0.87 (0.08)	1.01 (0.10)	0.79 (0.08)	1.00 (0.10)	1.00 (0.10)
7	1.00 (0.09)	0.96 (0.09)	1.01 (0.10)	0.93 (0.10)	1.00 (0.10)	1.00 (0.10)
8	1.00 (0.09)	0.63 (0.06)	1.01 (0.12)	0.68 (0.08)	1.00 (0.13)	1.00 (0.12)
9	1.00 (0.09)	0.91 (0.08)	1.01 (0.12)	0.92 (0.09)	1.00 (0.13)	1.00 (0.12)
10	1.00 (0.09)	0.49 (0.06)	1.00 (0.17)	0.68 (0.11)	1.00 (0.20)	1.00 (0.18)
11	1.00 (0.09)	0.66 (0.07)	1.00 (0.17)	0.79 (0.11)	1.00 (0.20)	1.00 (0.18)
12	1.01 (0.09)	0.88 (0.08)	1.01 (0.17)	0.92 (0.12)	1.00 (0.20)	1.00 (0.18)
13	1.00 (0.09)	0.75 (0.07)	0.90 (0.12)	0.76 (0.08)	0.87 (0.12)	0.88 (0.11)
14	1.00 (0.09)	0.74 (0.07)	0.70 (0.10)	0.72 (0.07)	0.67 (0.10)	0.67 (0.09)
15	1.00 (0.09)	0.76 (0.07)	0.39 (0.07)	0.74 (0.07)	0.38 (0.07)	0.38 (0.07)

Abbreviations: Exclude: exclusion of treated individuals from the analysis; *IPW* inverse (treatment) probability weighting; *IPW*_*trunc*_ IPW with weight truncation at 98th percentile

**Table 4:** Estimated discrimination in the validation set (c-index) across fifteen different simulated scenarios

Scenario	Method					
	Reference: untreated	Ignore treatment	Exclude treated	IPW	IPW, exclude	IPW_trunc_ exclude
1	0.67 (0.02)	0.63 (0.02)	0.65 (0.03)	0.66 (0.03)	0.66 (0.05)	0.65 (0.04)
2	0.67 (0.02)	0.66 (0.02)	0.67 (0.03)	0.66 (0.02)	0.67 (0.03)	0.67 (0.03)
3	0.67 (0.02)	0.59 (0.03)	0.65 (0.03)	0.64 (0.03)	0.66 (0.05)	0.65 (0.04)
4	0.67 (0.02)	0.59 (0.03)	0.60 (0.04)	0.59 (0.08)	0.57 (0.15)	0.60 (0.05)
5	0.67 (0.02)	0.62 (0.02)	0.65 (0.03)	0.66 (0.03)	0.67 (0.03)	0.66 (0.03)
6	0.67 (0.02)	0.64 (0.02)	0.65 (0.03)	0.66 (0.03)	0.66 (0.03)	0.66 (0.03)
7	0.67 (0.02)	0.66 (0.02)	0.65 (0.03)	0.67 (0.03)	0.67 (0.03)	0.66 (0.03)
8	0.67 (0.02)	0.60 (0.03)	0.65 (0.03)	0.66 (0.03)	0.66 (0.05)	0.65 (0.04)
9	0.67 (0.02)	0.65 (0.02)	0.65 (0.03)	0.66 (0.03)	0.66 (0.05)	0.65 (0.04)
10	0.67 (0.02)	0.61 (0.03)	0.65 (0.05)	0.66 (0.05)	0.66 (0.08)	0.65 (0.06)
11	0.67 (0.02)	0.64 (0.03)	0.65 (0.05)	0.66 (0.05)	0.66 (0.08)	0.65 (0.06)
12	0.67 (0.02)	0.66 (0.02)	0.65 (0.05)	0.66 (0.04)	0.66 (0.08)	0.65 (0.06)
13	0.66 (0.02)	0.63 (0.02)	0.63 (0.03)	0.65 (0.03)	0.64 (0.05)	0.63 (0.04)
14	0.65 (0.02)	0.63 (0.02)	0.60 (0.04)	0.62 (0.03)	0.61 (0.04)	0.60 (0.04)
15	0.62 (0.02)	0.61 (0.03)	0.57 (0.05)	0.58 (0.03)	0.57 (0.05)	0.57 (0.05)

Abbreviations: Exclude: exclusion of treated individuals from the analysis; *IPW* inverse (treatment) probability weighting; *IPW*_*trunc*_ IPW with weight truncation at 98th percentile

Results were derived from development and validation sets of 1000 individuals. Performance estimates are the means (and standard deviations) of the distribution of O:E ratios from 10,000 simulation replicates. See Table 2 for details of the scenarios.

Results were derived from development and validation sets of 1000 individuals. Performance estimates are the means (and standard deviations) of the distribution of c-indexes from 10,000 simulation replicates. See Table 2 for details of the scenarios.

### Ignore treatment

Ignoring the effects of treatment resulted, as expected, in predicted risks that were always greater than the observed outcome frequencies, suggesting poor model calibration in all scenarios. This was exacerbated in non-randomised settings, in which there appeared to be greater mis-calibration in high-risk individuals. When treatment allocation was non-random, ignoring treatment led to an underestimation of the c-index by up to 0.08 (scenario 3), whereas the c-index did not noticeably change in the RCT scenario. As expected, when either the effectiveness of treatment or the proportion of individuals treated increased, both the O:E ratio and c-index were more severely underestimated.

### Method 1: Exclude treated individuals

Excluding treated individuals resulted in calibration measures that appeared to reflect those of the untreated target population in most scenarios. However, as Fig. 3 shows, use of this approach when treatment allocation is dependent on an individual’s risk results in a loss of information about calibration in high risk individuals. When treatment allocation was random (scenario 2), this approach yielded a correct estimate of the c-index. As treatment allocation became increasingly associated with an individual’s risk across scenarios, this method yielded lower estimates for discrimination than observed in the untreated set, due to the selective exclusion of high-risk individuals, and consequently a narrower case-mix. The estimates of the c-index and O:E ratio were constant as the treatment effect and proportion treated changed across scenarios 5–12. In the presence of a strong unmeasured predictor of the outcome associated with treatment use (scenarios 14–15), exclusion of treated individuals resulted in an underestimation of the performance of the model. In addition, in all scenarios the precision of estimates of both the O:E ratio and c-index decreased due to the reduction in effective sample size.

### Method 2: Inverse probability weighting

Across all scenarios, IPW alone did not improve calibration, compared to when treatment was ignored, whereas IPW followed by the exclusion of treated individuals provided correct estimates for calibration. IPW alone or followed by the exclusion of treated individuals improved estimates of the c-index in all scenarios where the assumptions of positivity and no unobserved confounding were met. In scenario 4, where treatment allocation was determined by a strict risk-threshold and thus the assumption of positivity was violated, IPW was ineffective, and resulted in the worst estimates of discrimination across all methods. In addition, the extreme weights calculated in scenario 4 led to very large standard errors. In scenarios 13–15, the presence of an unobserved confounder led to the failure of IPW to provide correct estimates of the c-index. Weight truncation at the 98% percentile increased precision, but was less effective in correcting of the c-index for the effects of treatment.

## Discussion

We showed that when externally validating a prognostic model that was developed for predicting “untreated” outcome risks, treatment use in the validation set may substantially impact on the performance of the model in that validation set. Treatment use is problematic, if ignored, regardless of how treatment has been allocated, though more challenging to circumvent when non-randomized. While the risk-lowering effect of treatment seems to have little effect on model discrimination in randomised trial data, the model will appear to systematically over-estimate risks (mis-calibration). This effect worsens with greater dependency of treatment use on patient characteristics (e.g. baseline risk).

We present simple methods that could be considered when attempting to take the effects of treatment use into account. While the use of IPW in prediction model research is uncommon, the rationale behind using IPW in settings with non-randomized treatments is motivated by its use to remove the influence of treatment on causal (risk) factor-outcome associations [27, 28]. Although the use of IPW prior to the exclusion of treated individuals is a promising solution in data where treatments are non-randomly allocated, it should not be used when there are severe violations of the underlying assumptions, e.g. in the presence of non-positivity (where some individuals had no chance of receiving treatment), or when there is an unobserved confounder, strongly associated with both the outcome and treatment use. There is thus a need to explore alternative methods to IPW to account for the effects of treatment use when validating a prognostic model in settings with non-random treatment use.

Although the results of our simulations support the expected behaviour of the methods described in section 2.2, some findings warrant further discussion. First, although excluding treated individuals when treatments use is non-random theoretically results in incorrect estimated of model performance, in our simulations, the impact on model discrimination was small in most scenarios. However, when the association between an individual’s risk profile and the chance of being treated increased (scenario 4), the selection bias due to excluding treated individuals resulted in a large decrease in the c-index, as expected. Second, in simulated scenarios in which an unobserved confounder of the treatment-outcome relation was present, the performance of the model greatly decreased after excluding treated individuals, with or without IPW. This is likely due to the selective exclusion of individuals with a high value for the strongly predictive unobserved variable. This results in a narrower case-mix distribution, and consequently lower model discrimination, as well as mis-calibration due to the exclusion of a strong predictor of the outcome.

While it is unclear to what extent treatment use has affected existing prognostic model validation studies, findings from a systematic review of cardiovascular prognostic model studies indicate that changes in treatment use after baseline measurements in a validation study are rarely considered in the analysis [29]. While a number of studies excluded prevalent treatment users from their analyses, the initiation of risk-lowering interventions, such as statins, revascularization procedures and lifestyle modifications during follow-up was not taken into account. An equally alarming finding was that very few validation studies even reported information about treatment use during follow-up, raising concerns over the interpretation of the findings of these studies. Based on the findings of the present study, we suggest that information about the use of effective treatments both at the study baseline and during follow-up should be reported in future studies.

It must be noted that not all prediction model validation studies require the same considerations for treatment use. Although we have discussed prognostic models used for predicting the risk of an outcome without treatment, sometimes prognostic models are developed for making predictions in both treated and untreated individuals. If, for example, the treatments used in the validation set are a part of usual care, and are present in the target population for the model, then differences in the use of these treatments between the development and validation sets should be viewed as a difference in case-mix and not as an issue that we need to remove. Furthermore, if the model adequately incorporates relevant treatments (e.g. through the explicit modelling of treatment use), differences in treatment use between the development and validation sets can again be viewed as a difference in case-mix. In the event that treatments have not been modelled (e.g. because a new treatment has become readily available since the development of the model), the model could be updated through recalibration, or better yet by including a term for treatment in the updated model, leading to a completely new model, which in turn would require validation. Researchers must therefore first identify which treatments used in a validation data set could bias estimates of model performance, if ignored.

There are limitations to the guidance that we provide. First, we do not present a complete evaluation of all possible methods across a range of different settings, which would require at least an extensive simulation study. We argue, however, that the logical argumentation provided for each method forms a good starting point for further investigation. Furthermore, the list of methods that we present is by no means exhaustive and we encourage the consideration and development of new approaches for more complex settings, such as time-to-event settings, and where limited sample sizes pose a challenge. Second, we assumed for simplicity that a model has been developed in an untreated data set. In reality, it is likely that a model has been developed also in a partially treated set. The considerations for validation then remain the same, but it should be noted that failure to properly account for the effects of treatment in the development of a model can lead to a model that underestimates untreated risks [13]. Third, for simplicity we considered single point treatments in our simulated examples. Patterns of treatment use in reality are often complex, with individuals receiving multiple non-randomized treatments, even in RCTs. Finally, we also recognize that while this paper discusses the validation of prognostic models, the same considerations for treatment use can, in some circumstances, be relevant to diagnostic studies (i.e. where treatment between index testing and outcome verification could lead to similar- and even more serious- problems).

## Conclusion

When validating a previously developed prediction model for predicting risks without treatment in another data set, failure to properly account for (effective) treatment use in that validation sample will likely lead to poor performance of the prediction model and thus measures should be taken to remove the effects of treatment use. When validating a model with data in which treatments have been randomly allocated, simply excluding treated individuals is sufficient, at the cost of a loss of precision. In observational studies, where treatment allocation depends on patient characteristics or risk, inverse probability weighting followed by the exclusion of treated individuals can provide correct estimates of the actual performance of the model in its target population.

## Additional file

Additional file 1: R code for data generation, methods and analyses (“codefile.R”). (R 6 kb)

## Abbreviations

CVD: Cardiovascular disease
IPW: Inverse probability weighting
LP: Linear predictor
O:E: Observed:expected
OR: Odds ratio
PS: Propensity score
RCT: Randomized trial
ROC: Receiver operator characteristic
SD: Standard deviation
